# Acceptability of the community-level provision of Sayana® Press by medical and nursing students in Kinshasa, Democratic Republic of the Congo

**DOI:** 10.1016/j.contraception.2017.05.014

**Published:** 2017-09

**Authors:** Jane T. Bertrand, Paul Bukutuvwidi Makani, Julie Hernandez, Pierre Akilimali, Bitshi Mukengeshayi, Saleh Babazadeh, Arsene Binanga

**Affiliations:** a Tulane School of Public Health and Tropical Medicine, 1440 Canal St., Suite 1900, New Orleans, LA 70112, USA; b Institut Supérieur de Statistiques, Kinshasa, DRC; c Kinshasa University School of Public Health, DRC; d Action Santé et Dévéloppement, Kinshasa, DRC; e Tulane International LLC, Kinshasa, DRC

**Keywords:** Sayana® Press, Community-based distribution, Injectables, Kinshasa, DRC, Acceptor study, Medical & nursing students

## Abstract

**Objectives:**

The objectives were to assess acceptors' attitudes toward Sayana® Press as a method and toward the mechanism of community-based distribution by medical and nursing (M/N) students, known locally as “DBCs,” in Kinshasa, Democratic Republic of the Congo, and to evaluate the experience of these DBCs.

**Study design:**

In 2015, surveys were conducted among (1) acceptors of Sayana® Press on the day of the initial injection, (2) these same acceptors 3 months later and (3) the DBCs providing community-based services. The analysis was descriptive and involved no significance testing.

**Results:**

Acceptors of Sayana® Press expressed high levels of satisfaction with the method, despite some pain experienced at injection and subsequent side effects. Although most were satisfied with the counseling and services received from the DBCs, less than one third realized that the providers were M/N students. The DBCs expressed satisfaction in serving as community-based distributors; more than 95% would recommend it to others. Their primary complaints were lack of remuneration, stockouts and need for greater supervision.

**Conclusions:**

Consistent with results from previous pilot introductions of Sayana® Press in three African countries, clients were highly satisfied with Sayana® Press as a method. The reported preference for resupply at health centers may reflect a lack of client awareness that the DBCs administering methods near the health center were not in fact staff from the health center. The pilot served to gain acceptance for the use of M/N students in community-based distribution, paving the way for additional task-shifting pilots in Kinshasa.

**Implications:**

Sayana® Press represents a promising new method for increasing access to modern contraception in low-income countries. The Kinshasa experience is the first to test the use of medical and nursing students as providers at the community level. The study reports high levels of satisfaction on three counts: acceptors of the contraceptive method, acceptors of the mode of service delivery, and DBCs in their role as providers of contraception at the community level. However, many clients were not aware that the DBCs were students. The study represents an important contribution to the literature on task-shifting, especially in a country with chronic shortages of healthcare personnel.

## 1 Introduction

Sayana® Press emerged in 2011 as a promising new option that could increase access to contraception, especially at the community level in low-resource countries [1]. The product is a subcutaneous formulation of the intramuscular depot injectable contraceptive medroxyprogesterone acetate (DMPA-IM), available in the prefilled Uniject™ injection system [2]. Sayana® Press has great potential to increase contraceptive use worldwide among women who need an effective, reversible and discrete method [3].

Because of the ease of application, Sayana® Press offers the potential for task-shifting — from clinically trained medical personnel to community-based distributors and to self-injection [4]. A WHO consultative group recognized that a “compact, auto-disposable device” like Uniject™ can be used by lay health workers [5].

The few pilot studies conducted to date have shown a high level of acceptability among Sayana® Press users in community settings. Research among injectable users in Senegal and Uganda demonstrated that Sayana® Press was an acceptable alternative to DMPA-IM [2]. In Uganda, 84% of women choose Sayana® Press over DMPA-IM after trying both methods over a 6-month period [2]. Reasons given for this preference included the shorter needle, shorter application time with the prefilled injection and milder side effects. In terms of self-injection, women in a focus group study conducted in Ethiopia expressed a willingness to obtain Sayana® Press via self-injection provided that they had received adequate training to do so [6]. Although Sayana® Press has potential for increasing contraceptive uptake worldwide [1], [3], the available evidence of the feasibility and acceptability of application at the community level is still very limited.

Task-shifting (i.e., the provision of injectable contraceptives by community health workers) has yielded positive results in different African countries [7], [8]. However, in Kinshasa, Democratic Republic of the Congo (DRC), only doctors and nurses are authorized to give injections, which ostensibly precluded the possibility of community-level provision of Sayana® Press. However, the pilot introduction took advantage of one exception to the rule: that medical and nursing (M/N) students are authorized to give injections under the supervision of a clinical supervisor.

Modern contraceptive prevalence is very low in Kinshasa: 23% among married women; and 23% have an unmet need for contraception as of 2016 [9], reflecting both limited access to and fragile demand for contraception in this population. Thus, the government, donors and implementing organizations are actively searching for strategies that will increase access and contraceptive uptake. Delivering services outside fixed clinics reduces certain barriers of access; testing such strategies is a priority to the family planning community.

The objectives of this research were to assess acceptors' attitudes toward Sayana® Press as a method and toward the mechanism of community-level distribution by M/N students in Kinshasa, DRC, and to evaluate the experience of the students as community-based distributors. All subsequent mentions of “acceptors” refer to acceptors of Sayana® Press.

## 2 Materials and methods

### 2.1 The pilot intervention

A total of 10 institutes participated in the pilot: four medical schools (with students ranging from 18 to 24 years old) and six nursing institutes (whose students were mostly 16–20 years old). Each institute recruited approximately 13 students and 1 focal point (member of the teaching faculty authorized to serve as supervisor during the training and fieldwork). The organizers required that the institutes recruit students from advanced levels (grades) but left any other selection criteria to the schools. The 135 M/N students were called “DBCs” for *distributeurs à base communautaire* or community-based distributors. All DBCs underwent a 7-day training course which covered contraceptive technology (full range of methods), use of eligibility checklists (endorsed by Ministry of Health), counseling techniques, referrals, home visits and service statistics reporting. They also participated in a field exercise that included counseling and provision of methods.

The pilot took place primarily during “campaign days” in 5 of Kinshasa's 35 health zones (HZs): Kintambo, Kinsenso, and Lingwala (urban); Nsele and Makulu II (rural). The organizers contacted the health authorities of these HZs, scheduled the campaign day and enlisted community agents to invite women interested in learning about contraception and receiving free contraception to attend. On the campaign day, 15–20 M/N students arrived at the location (often adjacent to a health center) to provide group and individual counseling, deliver one of four methods onsite (pills, condoms, CycleBeads®, or Sayana® Press) to eligible clients and refer others to health centers for methods provided only by clinical personnel. DBCs also distributed methods during home visits and on-campus, but these clients were excluded from this research.

### 2.2 Data collection

#### 2.2.1 Initial acceptor survey (July–August 2015)

Fifteen female interviewers with previous experience in contraceptive surveys received training on the questionnaire content and survey procedures. This interviewer team accompanied the DBCs on campaign days. (Only) Clients who accepted Sayana® Press were enrolled in the study. The interviewer explained the study objectives to the acceptor and obtained informed consent. Because the number of acceptors was unknown in advance, interviewers were not always available immediately to conduct the interview. Ideally, we would have interviewed the universe of acceptors on the designated campaign days. Because some acceptors were unable or unwilling to stay for the interview, we reverted to a convenience sample.

#### 2.2.2 Follow-up survey of acceptors (October–November 2015)

Three months later, the teams of DBCs and interviewers revisited the same HZs to provide follow-up injections (or other methods) to interested clients and to interview the previous acceptors of Sayana® Press for a follow-up survey.

#### 2.2.3 DBC survey (November 2015)

After interviewer training, a second team of 6 male interviewers administered a questionnaire to DBCs onsite at the 10 participating M/N training institutes.

Interviewers in all three surveys collected data using smartphones programmed with the OpenDataKit application. The automatic data transfer to a secure online server (FormHub) allowed study personnel to review the data in real time and instruct interviewers of any midcourse corrections. Most questions were multiple choice, but questions such as complaints about the program allowed for write-in answers. No respondents received compensation for their participation in the surveys.

The data were analyzed using STATA. This descriptive analysis required no significance testing.

## 3 Results

### 3.1 Initial acceptor and 3-month follow-up surveys

A total of 8435 women accepted a contraceptive method from the DBCs during the pilot campaign days: 52% pill, 26% CycleBeads and 23% Sayana® Press. (Condoms were distributed but not considered the primary method for any user.)

A total of 374 acceptors of Sayana® Press participated in the initial interview. The median age was 25 years. These women had a slightly higher level of education than the general population, with 60% (vs. 47%) having at least completed secondary school [10]. Over half (56%) had no remunerated work. The majority (65%) were married or lived in union, and almost all of them (95%) had children (median of 3).

Of these acceptors, 52% had never used a contraceptive before and 30% had only used traditional methods. Among the minority who had ever used a modern method, condoms, injectables and pills were most frequent. Overall, only 14% of initial acceptors had ever used injectables.

Previous contact with a DBC was rare: only 8% had received a visit, and only 2% had visited a DBC to obtain contraception.

Among the 30% of acceptors who expressed some concerns about Sayana® Press, the reasons were fear of infertility, pain during or after the injection, effectiveness and safety. However, 75% intended to select Sayana® Press as their method “next time.” Over half (56%) of these women would prefer to get their method at a health facility.

Of the 374 initial acceptors, 252 (67%) were interviewed 3 months later: 220 when they returned for their second visit and 32 through follow-up. Among these women, 58% reported experiencing some side effects after receiving their first Sayana® Press injection: heavy bleeding (for 36% of them), no period (31%), irregular periods (31%) and abdominal pain (12%). By contrast, 42% experienced no side effects. Among previous users of DMPA-IM, 83% reported similar or fewer side effects; most were undeterred from continuing with Sayana® Press.

Among the 234 women interviewed on Round 2 who had already had their follow-up appointment, 93% chose to receive a second injection of Sayana® Press (with 4% choosing another contraceptive method and 3% choosing no method). The reasons for continuing with Sayana® Press included ease of use (65%), few side effects (44%), less painful than other methods (21%), easy to hide (15%) and no need to go to the health center (1%) (multiple responses allowed). Among 31 initial acceptors who chose not to continue with Sayana® Press, the main reasons were side effects/health issues (*n*=23) and concerns about safety (*n*=5). In both surveys, approximately 90% would recommend the product to a friend or a family member.

In the initial survey, 91% of acceptors reported satisfaction with the services received, yet 56% preferred to return to the health center for their next injection. This finding led the researchers to question whether the acceptors knew that providers were students and not health center personnel. In the follow-up survey, we explicitly asked if they knew. Less than one third of acceptors (30%) were fully aware that the DBC providing them with Sayana® Press was a student. Among the 74 clients who did not know, almost all nonetheless reported being “very comfortable” (97%) with having the DBC perform the injection. Moreover, 82% of all acceptors who came for their second appointment indicated being “very” or “somewhat ready” to receive their next Sayana® Press injection from a DBC, and 85% indicated that they would recommend receiving Sayana® Press from a DBC to their friends.

### 3.2 Survey of DBCs

A team of six male interviewers interviewed DBCs onsite at the 10 participating M/N training institutes approximately 6 months after the initial DBC training. Of 135 DBCs listed as participating in the pilot, 124 (92%) were successfully interviewed. The remaining 11 had graduated, moved or dropped out of school for financial reasons. Of the total, 53% were nursing students, 72% were female, and median age was 22 years old. One in five were married or in union; 15% had at least one child. Most (84%) did not have a paying job, although 96% had a cell phone.

Most (84%) felt that the training had prepared them very well to be a DBC.

A referral system had been established for DBCs to refer women experiencing side effects or opting for long-acting methods to visit a nearby healthcare facility. Most DBCs correctly answered hypothetical questions regarding referral to this site for other methods (85%) or for side effects (83%). However, only 23% had referred a client to a health facility. Less than half (47%) knew a health facility in their area that carried Sayana® Press and could counsel women who had side effects. Of this limited number (*n*=58), less than half (47%) knew someone who worked at one of these facilities.

Regarding adequacy of contraceptive resupply, more than half (52%) of the DBCs experienced at least one stockout of contraceptives during the Sayana ® Press pilot, most frequently of condoms and Sayana® Press. Although four in five reported the stockout to their supervisor, a quarter were never restocked.

The DBCs reported the overall reaction of the community toward their distribution of contraceptives as favorable (96%). In the few cases of opposition, the complaints concerned contraception in general, not the specific activity of the DBCs.

Over 90% were satisfied with their role as DBCs. Three main sources of satisfaction — stated by at least half the DBCs — were the opportunity to gain technical experience related to their career, to improve their interpersonal skills as healthcare providers and to contribute to the well-being of the community.

Despite their satisfaction, almost half of the DBCs had one or more complaints: lack of financial compensation (44%); frustration over negative rumors or resistance from the community (24%); and inadequate support from their supervisor or HZ administration, especially with regard to stockouts and referral issues. Of all DBCs, 97% would encourage other M/N students to become DBCs.

## 4 Discussion

Based on the published literature, the DRC is the first country to have used M/N students as community-based distributors of Sayana® Press. This model emerged in response to the restrictive legislation in the DRC whereby only doctors and nurses (and M/N students under supervision of a clinical supervisor) are authorized to give injections.

Consistent with introductory pilot studies of Sayana® Press in other Sub-Saharan African countries (Uganda, Senegal, Ethiopia) [2], [6], this research demonstrated a high level of acceptability among women who selected Sayana® Press, many of whom had not previously used modern contraception. Acceptors perceived the method as easy to use, effective and discrete. The concerns over Sayana® Press mirrored those of other contraceptives (fear of side effects including infertility, effectiveness and safety) and were not specific to this method. In short, the study showed high levels of client satisfaction with the method; 9 in 10 acceptors would recommend it to a friend.

Similarly, acceptors expressed relatively high levels of satisfaction with the provision of the method by DBCs. Yet the majority in both rounds would prefer to receive their next injection at a health center rather than “in the community” (meant to imply “in their neighborhoods or at home”). At first glance, this result seemed to contradict the expressed high levels of satisfaction with the services received; however, several explanations seem plausible. Because the counseling and delivery of methods on campaign days often took place adjacent to a health center, clients may have assumed that the DBCs formed part of the clinic staff; thus, their “preference” was to return to this same location for future injections. They may not have realized that this special type of campaign day would occur a second time. For this population, the local health center is their only source of health services (except for major accidents or illness requiring hospitalization); thus, the response “health center” may be an ingrained reflex. Finally, in a population where access to contraception (especially free contraception) is limited, interested clients may have been far more focused on obtaining their method than on the cadre of the provider giving the injection.

The study identified several challenges in using M/N students as DBCs: scheduling conflicts with their academic program, competing demands on their time, attrition when they graduate. Yet the retention rate was high: 92% were still participating and available for the interview 6 months posttraining. The pilot confirmed the assumption that students would value this type of community-based activity for improving their skills at service delivery and client interaction. Moreover, the majority expressed satisfaction in contributing to improved well-being of their community.

However, a number of issues emerged, which have been noted in other DBC programs operating in weak health systems [11]: lack of financial compensation despite difficult working conditions, gaps in the supply and routine information systems, frustrations generated by community opposition vis-à-vis contraception, and perceived lack of supervision and support from supervisors and HZs. Issues of contraceptive logistics (including stockouts, storage and waste disposal strategies) stem from systemic issues beyond the scope of Sayana® Press introduction, as noted elsewhere [12]. If left unresolved, these issues can threaten the effectiveness of the program and lead to burnout among the providers in question.

The study had several limitations. Acceptors were recruited using convenience sampling rather than a random sample of all women recruited through this program; the potential bias is unknown but may have yielded a sample of more favorable clients. The locations used for service delivery on campaign days were often crowded, making it hard to ensure that the acceptor was interviewed in private. Almost one third (33%) of Round 1 acceptors were lost to follow-up due to incorrect addresses or phone numbers. Thus, the Round 2 sample was likely biased toward those women who had a positive experience with Sayana® Press. Acceptors may not have understood the question regarding preferred location for their next injection.

The DBC survey attempted to interview all who had participated in the pilot. These students cannot be considered representative of other M/N students in Kinshasa; it is likely that more motivated students were recruited over less motivated ones. The study did not track the individual performance of the M/N students (in terms of volume of contraception distributed), and thus, we cannot compare their relative effectiveness of males versus females or medical versus nursing students as DBCs.

Kinshasa has had relatively little history of contraceptive provision by community health workers, in contrast to the other countries where Sayana® Press was piloted (e.g., Ethiopia, Senegal, Uganda) [2], [6]. This pilot not only legitimized the provision of Sayana® Press at the community-level by M/N workers, but it paved the way for further experimentation to increase access through community-based distribution, including the pilot introduction of self-injection of Sayana® Press and use of M/N students to provide Implanon NXT®.
